# Calcium-dependent protein kinase CPK32 mediates calcium signaling in regulating Arabidopsis flowering time

**DOI:** 10.1093/nsr/nwab180

**Published:** 2021-09-27

**Authors:** Xidong Li, Limei Chen, Li Yao, Junjie Zou, Jie Hao, Weihua Wu

**Affiliations:** State Key Laboratory of Plant Physiology and Biochemistry, College of Biological Sciences, China Agricultural University, Beijing 100193, China; State Key Laboratory of Plant Physiology and Biochemistry, College of Biological Sciences, China Agricultural University, Beijing 100193, China; Center for Crop Functional Genomics and Molecular Breeding, China Agricultural University, Beijing 100193, China; State Key Laboratory of Plant Physiology and Biochemistry, College of Biological Sciences, China Agricultural University, Beijing 100193, China; Syngenta Biotechnology China Co., Ltd., Beijing 102206, China; State Key Laboratory of Plant Physiology and Biochemistry, College of Biological Sciences, China Agricultural University, Beijing 100193, China; Biotechnology Research Institute, Chinese Academy of Agricultural Sciences, Beijing 100081, China; State Key Laboratory of Plant Physiology and Biochemistry, College of Biological Sciences, China Agricultural University, Beijing 100193, China; State Key Laboratory of Plant Physiology and Biochemistry, College of Biological Sciences, China Agricultural University, Beijing 100193, China; Center for Crop Functional Genomics and Molecular Breeding, China Agricultural University, Beijing 100193, China

**Keywords:** calcium signaling, CPK32, FCA, FLC, flowering time

## Abstract

Appropriate flowering time is critical for the reproductive success of plant species. Emerging evidence indicates that calcium may play an important role in the regulation of flowering time. However, the underlying molecular mechanisms remain unclear. In this study, we demonstrate that calcium-dependent protein kinase 32 (CPK32) regulates flowering time by affecting the alternative polyadenylation of *FLOWERING CONTROL LOCUS A* (*FCA*) and altering the transcription of *FLOWERING LOCUS C* (*FLC*), a central repressor of flowering time. The knockdown of *CPK32* results in an obvious late flowering phenotype and dramatically enhanced *FLC* transcription. CPK32 interacts with FCA, and phosphorylates the serine^592^ of FCA in a Ca^2+^-dependent manner. Moreover, the ratio of abundance of the *FCA* transcripts (*FCA-D* and *FCA-P*) changes significantly in the *cpk32* mutant, which subsequently affects *FLC* expression and consequently regulates floral transition. The present evidence demonstrates that CPK32 modulates flowering time by regulating *FCA* alternative polyadenylation and consequent *FLC* expression.

## INTRODUCTION

Flowering time is critical for flowering plant reproduction, species maintenance, adaptation and domestication, and is also a key element in crop breeding and variety selection because of its importance for crop growth vigor and yield [1,2]. Molecular analysis and genetic studies of flowering time regulation have made great strides in deciphering the regulatory mechanism of floral transition.

*FLOWERING LOCUS C* (*FLC*) is the core regulatory gene mediating flowering time in the autonomous and vernalization pathways [3,4]. Alternative polyadenylation (APA) generates mRNAs with distinct 3^′^ ends and has emerged as a pervasive regulatory mechanism in gene expression [5,6]. Several floral regulators modulate *FLC* expression through APA, such as the conserved RNA-binding proteins FLOWERING CONTROL LOCUS A (FCA) and FLOWERING LOCUS PA (FPA) [7–11]. Interaction between FCA and FLOWERING LOCUS Y (FY) is required for the efficient selection of the 3' end of *FCA* transcripts and *FLC* repression [9,12]. Integrative genome-wide analysis revealed that the heterogeneous nuclear ribonucleoprotein (hnRNP) A1-like protein 1 (HLP1) regulates flowering by modulating the APA of *FCA* [13]. Despite the wealth of knowledge about the FCA-FLC module in the autonomous pathway, little is known about the molecular mechanism that regulates the APA of *FCA* during flowering.

Plants have evolved sophisticated signaling cascades that perceive, integrate and respond to endogenous and external stimuli, thereby ensuring their survival and the proper timing of flowering for species maintenance. Calcium (Ca^2+^) is a ubiquitous second messenger in living organisms [14]. Previous reports have shown that the environmental factors that influence flowering time, such as light quality [15], day length, light intensity [16] and low temperatures [17,18], also affect the cytosolic Ca^2+^ concentration in Arabidopsis roots, leaves or seedlings.

Ca^2+^ signals are perceived and decoded by a series of Ca^2+^-binding proteins or Ca^2+^ transducers. Among calcium-binding proteins, Ca^2+^-dependent protein kinases (CPKs/CDPKs) are plant-specific protein kinases that participate in a variety of important physiological activities, including ion channel regulation [19,20], stomatal movements [21], cell expansion [22] and responses to pathogens [23]. In the present study, we present evidence for the involvement of CPK32 in flowering time regulation. The *CPK32* knockdown mutant *cpk32* exhibited an obvious late-flowering-time phenotype, accompanied by significant enhancement of *FLC* expression. It was further revealed that CPK32 interacts with FCA, and phosphorylates the serine^592^ of FCA in a Ca^2+^-dependent manner. As a result, the alternative splicing of *FCA* pre-mRNA is regulated, and in turn *FLC* transcription is regulated. The presented evidence demonstrates that CPK32 regulates *FCA* pre-mRNA levels to fine-tune flowering time through the autonomous pathway.

## RESULTS

### CPK32 is a positive regulator of plant flowering

**Figure 1.**
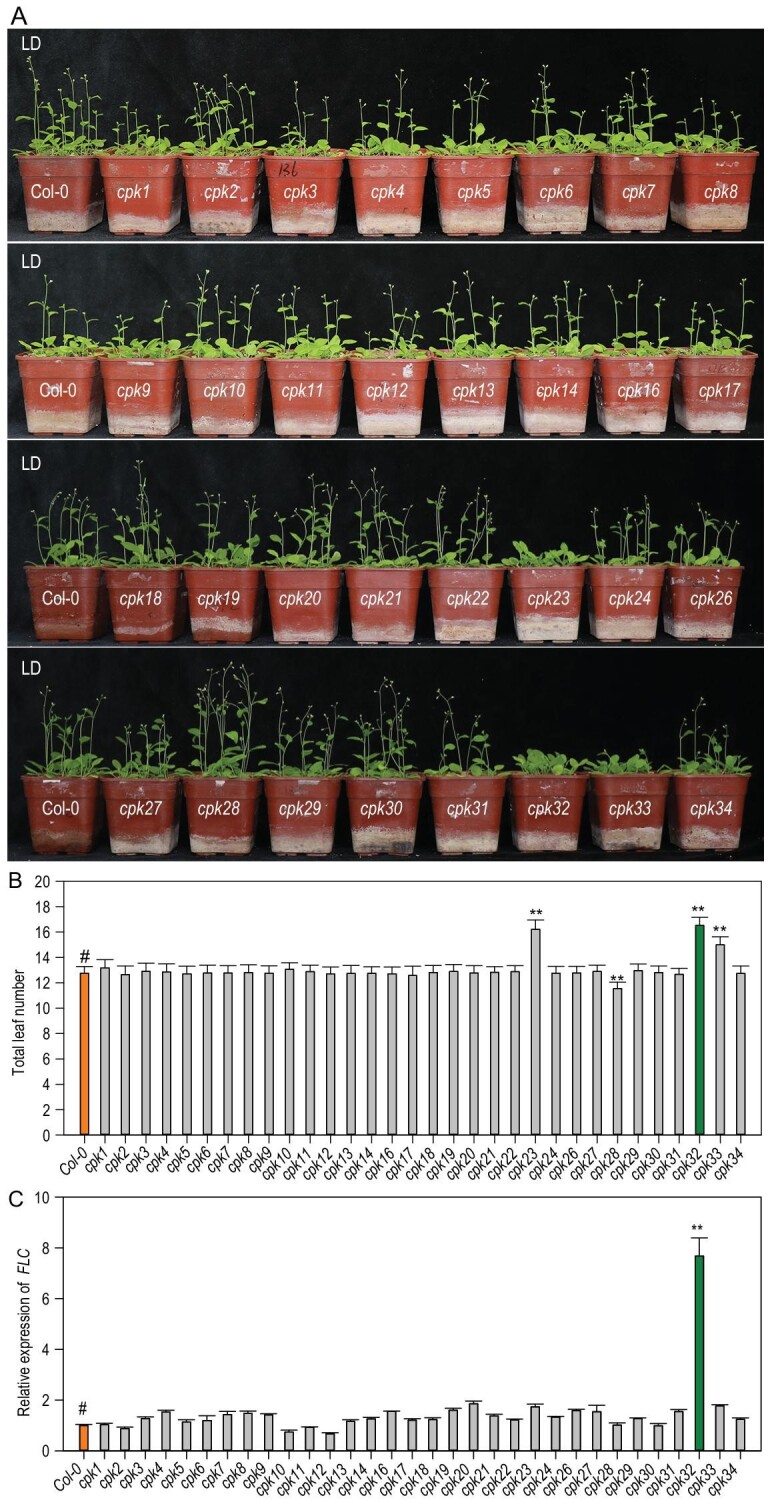
Effect of Arabidopsis *CPK* gene mutations on flowering time*.* (A) Flowering-time phenotype of Arabidopsis *cpk* mutants planted in LDs (16 h light/8 h dark). (B) Total leaf number per plant at flowering of *cpk* mutants planted shown in (A), *n *= 30. (C) *FLC* transcription in 7-day-old Arabidopsis *cpk* mutants, as determined by RT-qPCR assay, *n *= 3. (B) and (C) Experiments were repeated three times with similar results and the data are presented as the mean ± SE (Student's *t*-test; ^**^P < 0.01, and ‘#’ represents control).

The CPK family in the Arabidopsis genome includes 34 members, and they cluster into four subgroups based on protein sequence homology [24]. To investigate the potential function of CPKs in the floral transition in Arabidopsis, the flowering time phenotype of 32 *CPK* mutant lines (Supplementary Fig. S1 and Supplementary Table 1) were screened in long-day (LD) photoperiod conditions. At the time of bolting, the majority of *cpk* mutants showed a similar flowering time phenotype compared with the wild-type (WT) plants, except *cpk23*, *cpk32* and *cpk33* mutants, which displayed a late flowering phenotype, and the *cpk28* mutant (Salk_112540, an overexpression line), which displayed an early flowering phenotype (Fig. 1A). In Arabidopsis, flowering time is closely associated with leaf number, which is widely used to quantify the time of the floral transition [12,25]. At the time of flowering, these three late flowering mutants developed more leaves than the WT plants (Fig. 1B). CPK32 belongs to subgroup III, CPK23 and CPK33 belong to subgroup II, and CPK28 belongs to subgroup IV of the CPK super-family [24]. Their divergent homology suggests that they may regulate the flowering of Arabidopsis via different mechanisms. RT-qPCR experiments revealed that transcription of *FLC* was dramatically enhanced in the *cpk32* mutant, while *FLC* transcription was not changed in *cpk23*, *cpk28* and *cpk33* mutants (Fig. 1C). Considering FLC is a central repressor of flowering [3,4], further investigation was focused on characterizing CPK32 function in flowering time regulation.

**Figure 2.**
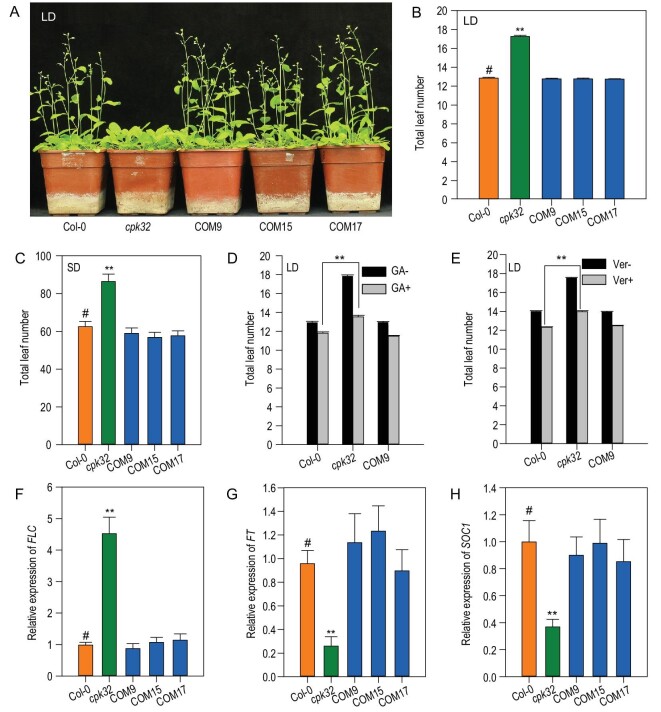
CPK32 regulates flowering time through the autonomous pathway. (A) and (B) Flowering-time phenotype of Col-0, *cpk32* and *cpk32* complementation lines planted in soil in LDs (A) and total leaf number per plant at flowering (B). Experiments were repeated three times with similar results and the data are presented as the mean ± SE, *n *= 100. (C) Total leaf number for Col-0, *cpk32* and the *cpk32* complementation lines grown in soil in SDs. The data are presented as the mean ± SE, *n* = 30. (D) and (E) Total leaf number at flowering for Col-0, *cpk32* and COM9 treated with (D) GA and (E) vernalization in LDs. Data are presented as the mean ± SE, *n *= 100. (F)–(H) RT-qPCR analysis of *FLC*, *FT* and *SOC1* transcription in Col-0, *cpk32* and *CPK32* complementation lines of 7-day-old LD-grown seedlings. Data are presented as the mean ± SE, *n *= 3. Student's *t*-test (#, control; ^**^, P < 0.01) was used to analyze statistical significance.

The *cpk32* mutant line used in our experiments is a knock-down allele (Supplementary Fig. S2). To validate that the late-flowering-time phenotype was caused by decreased *CPK32* expression, three independent complementation lines (COM9, COM15 and COM17) were generated (Supplementary Fig. S2C and D). All complementation lines displayed a flowering time similar to the WT in LD photoperiod conditions (Fig. 2A and B). These results confirmed that the decreased expression of *CPK32* in the *cpk32* mutant is responsible for its late-flowering-time phenotype and suggests that CPK32 may positively regulate the floral transition in Arabidopsis.

### Expression profiling of *CPK32*

To gain insight into the function of CPK32 during floral initiation, *CPK32pro : GUS* transgenic lines were used and the GUS staining showed that *CPK32* was expressed in embryos and emerged radicles, roots, floral organs, anthers and stigma (Supplementary Fig. S3). The results also showed that *CPK32* is expressed in the shoot apex (Supplementary Fig. S3D), indicating that CPK32 may play a role in floral initiation.

### CPK32 regulates flowering time through the autonomous pathway

Four major pathways regulating flowering time have been defined by classic genetics approaches in Arabidopsis [26,27]. To identify the pathway that CPK32 is involved in, the flowering-time phenotypes of the *cpk32* mutant were tested under different photoperiods, gibberellic acid (GA) treatment and vernalization treatments. Compared with LD conditions, short-day (SD) conditions extended the vegetative growth period of all lines indicated with their increased leaf number, and the *cpk32* mutant still flowered significantly later than the WT plants and the complementation lines (Fig. 2C). These results indicate that *cpk32* mutants respond to changes in photoperiod in a similar way to WT plants, except for their delayed flowering. The experiments conducted with GA and vernalization treatments in LD photoperiod conditions showed that all plants flowered earlier than their corresponding controls, indicating that the *cpk32* mutant is similar to WT in its response to GA and vernalization (Fig. 2D and E). Taken together, these data demonstrate that CPK32 does not promote floral transition through the photoperiod, GA and vernalization pathways but most likely through the autonomous pathway.

To confirm the involvement of CPK32 in the autonomous pathway, the *FLC* transcription in various lines was analyzed. As shown in Figs 1C and 2F, *FLC* transcriptional expression in the *cpk32* mutants was much higher than in WT plants, while the complementation lines showed similar transcription to the WT (Fig. 2F). Consistently with this, *FLOWERING LOCUS T* (*FT*) and *SUPPRESSOR OF OVEREXPRESSION OF CO 1* (*SOC1*), which are downstream targets of *FLC* in flowering-time control [28,29], were down-regulated in the *cpk32* mutant compared to the WT plants and the complementation lines (Fig. 2G and H).

**Figure 3.**
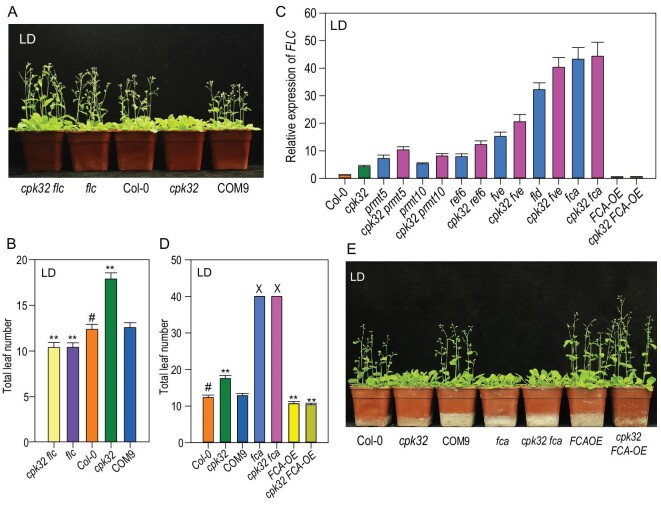
CPK32 regulates *FLC* expression through FCA. (A) Flowering-time phenotype of Col-0, *cpk32*, COM9, *flc* and *cpk32 flc* plants grown in soil in LDs. (B) Total leaf number for Col-0, *cpk32*, COM9, *flc* and *cpk32 flc* plants in LDs. The data are presented as the mean ± SE, *n* = 50. (C) RT-qPCR analysis of *FLC* transcription in the double mutants (i.e. *cpk32* crossed individually with six different mutants of the autonomous pathway). Data are presented as the mean ± SE, *n* = 3. (D) Total leaf number of *CPK32* and *FCA*-related materials grown in LDs. Experiments were repeated three times with similar results and the data are presented as the mean ± SE, *n *= 50 (‘X’ means that the counting was terminated after plants had produced over 40 total leaves before bolting). (E) Flowering-time phenotype of *CPK32-* and *FCA*-related plant materials grown in soil in LDs. Student's *t*-test (#, control; ^**^, P < 0.01) was used to analyze statistical significance.

To further confirm CPK32 involvement in floral transition through its effect on *FLC* expression, the *cpk32 flc* double mutant was generated (Supplementary Fig. S2E) and it showed the similar early flowering phenotype as the *flc* single mutant in LD photoperiod conditions (Fig. 3A and B). These results support our hypothesis that *CPK32* regulates flowering by repressing *FLC* expression.

### CPK32 regulates *FLC* expression through FCA

Previous reports have shown that a number of genes, such as *FCA*, *FPA*, *FY*, *FLK*, *LUMINIDEPENDENS*, *FLOWERING LOCUS D (FLD)*, *RELATIVE OF EARLY FLOWERING 6 (REF6)*, *FVE*, *PROTEIN ARGININE METHYLTRANSFERASE 5 (PRMT5)* and *PRMT10*, regulate flowering time via the autonomous pathway [27,30]. Among these genes, seven, including *FLK*, *FLD*, *FVE*, *FPA*, *FY*, *REF6* and *LUMINIDEPENDENS*, showed no obvious changes in their expression in the *cpk32* mutant compared to the WT (Supplementary Fig. S4A). *FLC* transcription in the double mutants *cpk32 prmt5*, *cpk32 prmt10*, *cpk32 ref6*, *cpk32 fve* and *cpk32 fld* was higher than that in corresponding single mutants, respectively, except the *cpk32 fca* double mutant (Fig. 3C and Supplementary Fig. S4B and C). *FLC* transcription in the *cpk32 fca* double mutant was nearly the same as that in the *fca* single mutant (Fig. 3C), suggesting that CPK32 may regulate *FLC* expression through FCA.

Previous reports have shown that FCA functions as a posttranscriptional regulator, and that the *fca* mutant has a late-flowering phenotype [7,25]. The *cpk32 fca* double mutant displayed the same phenotype as the *fca* single mutant in terms of flowering time, and *FCA-OE* and *cpk32 FCA-OE* plants (Supplementary Fig. S4D) showed lower *FLC* expression and earlier flowering-time phenotype (Fig. 3D and E). These results demonstrate that CPK32 and FCA act in the same pathway, and the upregulated expression of *FLC* in the *cpk32* mutant is most likely mediated by *FCA*.

### CPK32 interacts with FCA

**Figure 4.**
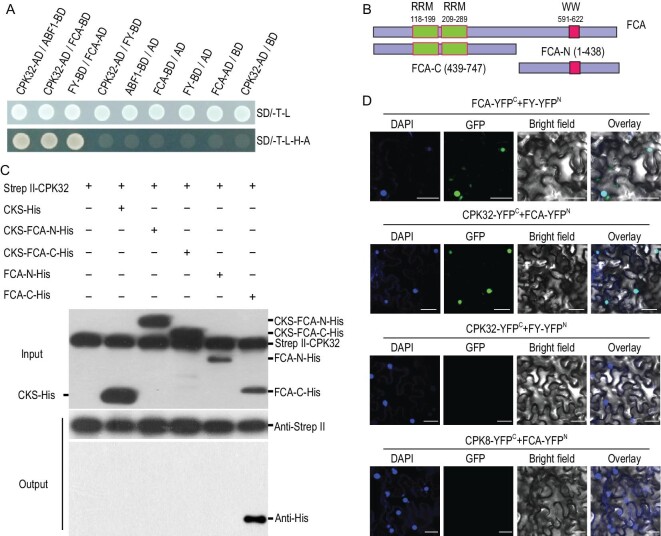
CPK32 interacts with FCA. (A) Interaction between CPK32 and FCA in yeast. CPK32-ABF1 and FCA-FY were used as positive controls and CPK32/pDEST32 as a negative control. (B) Diagram of FCA N-terminal and C-terminal truncations. (C) Pull-down assay for the interaction between CPK32 and FCA. The Strep II-CPK32 was used as a bait to pull down FCA-His fusion protein prey. Anti-Strep II and anti-His antibodies were simultaneous used to test the proteins in the input reaction. An anti-Strep II and anti-His antibody was separately used to blot the Strep II-CPK32 and the interacted truncated FCA protein. (D) BiFC assay of the CPK32 and FCA interaction in *N. benthamiana* leaves. The C-terminal half of YFP was fused to CPK32, and the N-terminal half of YFP was fused to FCA. FCA-YC/FY-YN was used as a positive control. DAPI was used to stain the cell nucleus. Scale bar: 50 μm.

As previous reports showed that FCA interacts with FY to downregulate *FLC* expression [12] and CPK32 interacts with Abscisic acid responsive element-Binding Factors (ABFs) [31], FCA-FY and CPK32-ABF1 were used as the positive controls in Yeast Two-Hybrid (Y2H) experiments to test the proposed interaction between CPK32 and FCA. Indeed, CPK32 strongly interacted with FCA (Fig. 4A), while FY, FLK and FVE had no interaction with CPK32 (Supplementary Fig. S5A). All the other seven subgroup III CPK members had no interaction with FCA (Supplementary Fig. S5B).

The protein pull-down assays further confirmed the interaction between CPK32 and FCA. The N-terminal of FCA containing two RNA recognition motif (RRM) domains and the C-terminal of FCA containing the tryptophan-tryptophan (WW) domain were purified (Fig. 4B). CPK32 interacted with the C-terminal region of FCA (FCA-C-His), but not with its N-terminal region (Fig. 4C). It should be noted that CPK32 did not interact with CKS-FCA-C-His, suggesting that CKS-tag, a 29 kD fragment, may weaken the interaction between CPK32 and the FCA-C terminal. In addition, bimolecular fluorescence complementation (BiFC) assays showed specific CPK32-FCA interaction signals in the nucleus (Fig. 4D). These results demonstrate that CPK32 directly interacts with FCA both *in vitro* and *in vivo*.

### CPK32 phosphorylates FCA in a Ca^2+^-dependent manner

**Figure 5.**
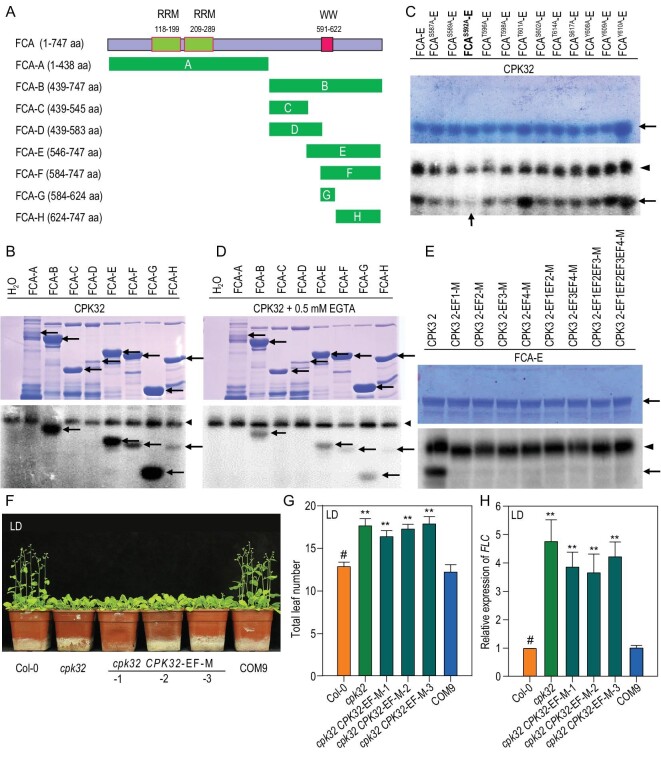
CPK32-mediated phosphorylation of FCA is dependent on Ca^2+^. (A) Diagram of FCA N-terminal and C-terminal truncations. The green bars represent different segments of FCA. (B) CPK32 phosphorylates FCA *in vitro*. Coomassie blue-stained recombinant proteins (FCA-A to FCA-H) are indicated by the arrows in the upper panel. In the lower panel, the arrowhead shows auto-phosphorylated CPK32, and the arrows show phosphorylated FCA variants. (C) Determination of phosphorylation site by CPK32 in FCA-E. As the potential phosphorylation sites, serine, threonine and tyrosine were mutated to alanine to mimic an unphosphorylated status. Coomassie blue-stained recombinant proteins of mutated FCA-E proteins are indicated by the arrow in the upper panel. In the bottom panel, the arrowhead shows auto-phosphorylated CPK32, and the arrow shows phosphorylated FCA-E. (D) EGTA weakens the phosphorylation mediated by CPK32. (E) CPK32-mediated phosphorylation of FCA is dependent on Ca^2+^ binding. Coomassie blue-stained recombinant proteins of FCA-E proteins are indicated by the arrow in the upper panel. In the bottom panel, the arrowhead shows auto-phosphorylated CPK32, and the arrow shows phosphorylated FCA-E. EF1, EF2, EF3 and EF4 denotes the first, second, third and fourth EF-hand respectively. (F) and (G) Flowering-time phenotype (F) and total leaf number (G) for Col-0, *cpk32*, COM9 and the EF-hand mutant complemented line grown in LDs. Data are presented as the mean ± SE, *n* = 50. (H) RT-qPCR analysis of *FLC* transcript levels in *cpk32,* the four EF-hand mutant complementation lines and COM9. Data are presented as the mean ± SE, *n* = 3. Student's *t*-test (#, control; ^**^, P < 0.01) was used to analyze statistical significance.

It was hypothesized that CPK32, as a protein kinase, may regulate FCA function by phosphorylation. Truncated FCA segments (Fig. 5A) were the substrates. Autoradiography showed that FCA-B, FCA-E, FCA-F, FCA-G and FCA-H fragments were phosphorylated by CPK32, and the strongest signal detected was in the FCA-G fragment (Fig. 5B), which mainly contains the WW domain required for FCA autoregulation [9]. The potential phosphorylation sites, including serine, threonine and tyrosine in the WW domain, were substituted with alanine to mimic an un-phosphorylation status for the phosphorylation assay. Autoradiography results showed that the phosphorylation signal of FCA^S592A^-E significantly decreased (Fig. 5C), suggesting that the serine^592^ is the target site of phosphorylation by CPK32.

Furthermore, FCA phosphorylation by CPK32 is Ca^2+^-dependent. Chelation of Ca^2+^ by addition of EGTA in the reaction medium resulted in weaker signals for all phosphorylated bands (Fig. 5D). The C terminus of  CPK protein contains a Ca^2+^-binding domain constituted by EF-hand motifs [24]. To determine whether CPK32-mediated phosphorylation of FCA is Ca^2+^ binding dependent, a series of single, double and multiple EF-hand motif mutations of CPK32 were generated as described previously [32], rendering these motifs unable to bind Ca^2+^. Using an FCA-E fragment as the substrate, phosphorylation signals were abolished for all CPK32 proteins with mutated EF-hands (Fig. 5E). The results further demonstrated that phosphorylation of FCA by CPK32 is Ca^2+^ dependent.

To further confirm correlation of CPK32-mediated Ca^2+^ signaling and flowering-time regulation, the transgenic lines (*cpk32 CPK32-EF-M*) with four EF-hands mutations [32] were generated (Supplementary Fig. S6) and used to test the flowering-time phenotype. As shown in Fig. 5F and G, the *cpk32 CPK32-EF-M* plants displayed the same delayed flowering-time phenotype as the *cpk32* mutant. Moreover, *FLC* expression in these EF-hand-mutated transgenic lines was similarly enhanced as in the *cpk32* mutant (Fig. 5H).

### CPK32 regulates the alternative polyadenylation of *FCA* by affecting FCA-FY interaction

**Figure 6.**
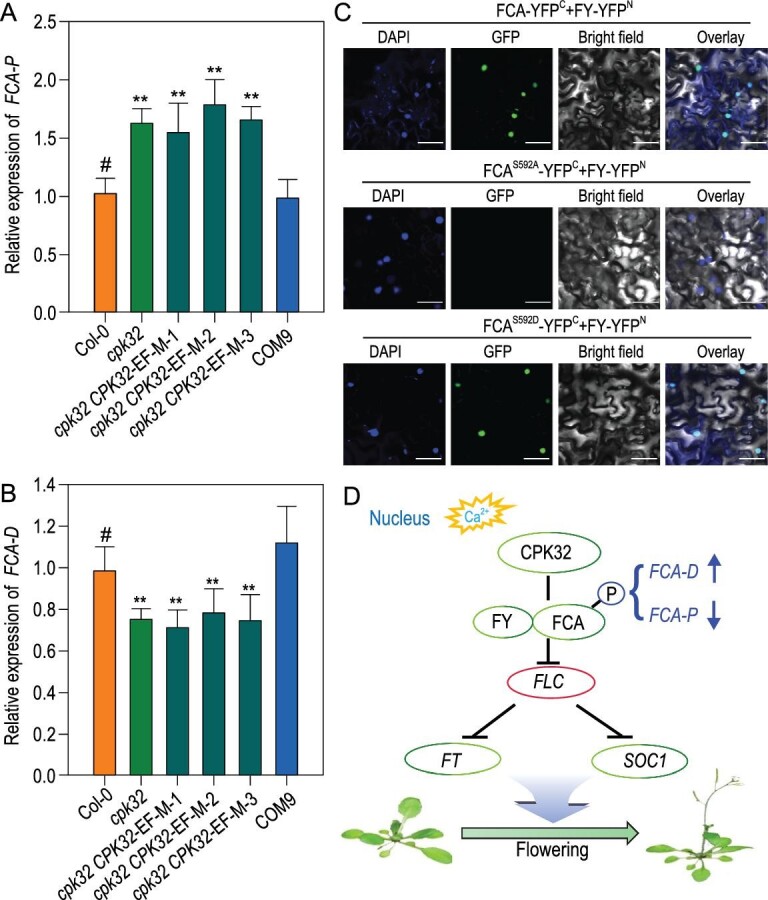
CPK32-mediated floral initiation via regulation of *FCA* alternative polyadenylation through the autonomous pathway. (A) and (B) RT-qPCR analysis of (A) proximal (*FCA-P*) and (B) distal (*FCA-D*) polyadenylated *FCA* transcripts in Col-0, *cpk32*, EF-hand mutant complementation lines and COM9. Experiments were repeated three times with similar results. Data are presented as the mean ± SE, *n* = 3. The asterisks indicate a significant difference relative to Col (Student's *t*-test, ^**^P < 0.01). (C) BiFC assay of the FY and mutated FCA interaction in *N. benthamiana* leaves. The C-terminal half of YFP was fused to FCA, and the N-terminal half of YFP was fused to FY. DAPI was used to stain the cell nucleus. Scale bar: 50 μm. (D) Working model for CPK32-mediated calcium signaling in regulation of Arabidopsis flowering time. Ca^2+^-activated CPK32 interacts with FCA and phosphorylates the WW domain of FCA in the nucleus. Phosphorylated FCA interacts with FY and in turn regulates the alternative splicing of *FCA* pre-mRNA. Changes in the ratio of functional FCA transcripts (*FCA-D*) to non-functional FCA transcripts (*FCA-P*) induces regulation of *FLC* expression and consequent flowering transition. The T-bar in the diagram represents negative regulation.

Previous studies have shown that FCA regulates its own expression by promoting premature cleavage and polyadenylation of its own pre-mRNA [7]. The *FCA* pre-mRNA can be alternatively spliced to form four different transcripts and only the *FCA-γ* can complement the late-flowering phenotype of the *fca* mutant [8]. CPK32 phosphorylated the WW domain, which is required for FCA autoregulation in a calcium-dependent manner (Fig. 5B, D and E). To test whether CPK32 plays a role in processing *FCA* mRNA, the distal (*FCA-D*, mainly *FCA-γ*) and proximal (*FCA-P*, mainly *FCA-β*) polyadenylation of *FCA* pre-mRNA were analyzed. Compared to the WT, the *cpk32* mutant had decreased levels of *FCA-D* transcripts and increased levels of *FCA-P* transcripts, while COM9 had approximately the same levels as WT (Fig. 6A and B). Obviously, disruption of *CPK32* expression in the *cpk32* mutant resulted in changes in the ratio of the *FCA-D* to *FCA-P* transcripts. Previous reports have shown that the functional *FCA-γ* (*FCA-D*) transcripts result in accelerated flowering [7,8], which together with the present results suggests that the late flowering-time phenotype of the *cpk32* mutant may result from the decreased functional distal polyadenylation *FCA* transcript.

The transcripts of *FCA-P* and *FCA-D* in EF-hand-mutated complementation lines did not resume at WT levels (Fig. 6A and B), demonstrating that CPK32 activity plays an important role in regulating *FCA* pre-mRNA processing. The WW domain is required for FCA autoregulation of RNA 3^′^ polyadenylation and mediates the interaction with FY, and this interaction significantly affects flowering time [12]. As shown in the BiFC assays, the FCA^S592D^, a persistent phosphorylated FCA form, showed an interaction with FY, while the FCA^S592A^, an unphosphorylated form of FCA, did not show the interaction between FCA and FY (Fig. 6C). These results suggest that CPK32 may regulate flowering time by maintaining the FCA-FY interaction via phosphorylation modification.

## DISCUSSION

In this study, we showed that CPK32 modulates flowering time by regulating *FCA* pre-mRNA processing in Arabidopsis. CPK32 interacts with FCA, phosphorylates the WW domain of FCA and regulates the APA of *FCA* transcripts (Figs 4–6). FCA plays an important role in alternative polyadenylation of antisense RNAs and 3^′^ end formation of *FLC* and its transcript [11,33]. As a result, *FLC* expression is suppressed and the inhibition on flowering transition is released (Fig. 6D).

Among *CPK* family members, the present data showed that *CPK32* is specifically involved in flowering-time control by repressing *FLC* expression (Fig. 1C). We also observed a late flowering-time phenotype for the mutant *cpk33* (Fig. 1A and B), which is consistent with previously reported results [34,35]. In addition to CPK32, CPK23, CPK28 and CPK33 are also involved in the regulation of flowering time (Fig. 1A and B), although these CPKs do not affect *FLC* expression (Fig. 1C). Further analysis of their functions would increase the understanding of the Ca^2+^ signaling mechanism’s role in regulation of flowering time.

CPKs have been identified as regulators involved in various physiological responses in plants [36]. Previous reports have shown that CPK32 can interact with ABFs and regulate ABA-responsive gene expression [32]. A genome-wide survey of CPK function in pollen tubes demonstrated that *CPK32* overexpression caused the formation of a bulge at the tip of the pollen tube [37]. CPK32 interacted with cyclic nucleotide-gated channel 18 (CNGC18) and promoted Ca^2+^ entry during the elevation phase of the Ca^2+^ oscillations that occurred during polar growth of pollen tubes [38]. In addition, CPK32 was one of the main regulators of nitrate-CPK-NIN-LIKE PROTEIN (NLP) signaling networks [39]. CPK32 also regulated the activity of ammonium transporter 1;1 (AMT1;1) [40]. It seems that CPK32 may be a multi-functional regulator and play an important role in integrating multiple signaling processes and optimizing plant growth and development.

In conclusion, this study has revealed that the Ca^2+^-dependent protein kinase CPK32 functions as a positive regulator of flowering time acting through the autonomous pathway, and this finding provides new insight into the complex regulatory network of flowering time and calcium signaling in plants.

## MATERIALS AND METHODS

Detailed descriptions of materials and methods are available as supporting information at *NSR* online.

## Supplementary Material

nwab180_Supplemental_Files
