# Supplementary material for: Calcium-dependent protein kinase CPK32 mediates calcium signaling in regulating Arabidopsis flowering time
Source: Natl Sci Rev. 2021 Sep 27;9(1):nwab180. doi: 10.1093/nsr/nwab180 (PMC8783668; doi:10.1093/nsr/nwab180)
Supplement: nwab180_Supplemental_Files [file nwab180_supplemental_files.zip › Methods_and_materials_V3_cleared.docx]

**MATERIALS AND METHODS**

**Plant Materials and Growth Conditions**

All *Arabidopsis thaliana* lines used in this study were in the Columbia-0 (Col-0) ecotype background. The T-DNA insertion lines *cpk32* (At3g57530, SALK_112665), *flc* (At5g10140, SALK_003346), and other *cpk* mutants (Supplemental Table 1) were obtained from the Arabidopsis Biological Resource Center (ABRC, <http://www.arabidopsis.org/abrc/>). *prmt5-1* (SALK_065814), *prmt10-1* (SALK_047046), *ref6-3* (SAIL_747A7), *fve-7* (SALK_013789), *fld-4* (SALK_015053), *fca-9* mutants were provided by Dr. Xiao-feng Cao (Institute of Genetics and developmental biology, CAS).

Seeds were surface sterilized with a mixed solution of NaClO (0.5%, v/v) and Triton X-100 (0.01%, v/v). Seeds were sown on Murashige and Skoog (MS) solid medium and allowed to germinate in a growth chamber at 22ºC in LDs (16 h at 120 μmol m^-2^ s^-1^ light/8 h dark) or SDs (8 h at 120 μmol m^-2^ s^-1^ light/16 h dark). After one week, seedlings were transferred to potting soil mixture (rich soil: vermiculite = 2:1, v/v) and maintained in LDs or SDs. The relative humidity was 70%.

For the GA treatment, seedlings were grown in Petri dishes containing MS medium and then transplanted to soil in LDs. Seedlings were sprayed with 100 μM GA_3_ three times per week until flowering occurred. Distilled water containing an equal concentration of ethanol was sprayed as a control. For the vernalization treatment, 7-day-old seedlings grown on plates were transferred to 4ºC in LDs for six weeks, and then moved to the soil grown at 22ºC. When the first flower bloomed and the stem length reached approximate 5 centimeters, the numbers of rosette and stem leaves were counted.

**Identification of T-DNA Insertion Mutants**

Genomic DNA was isolated from plants using an acetyl-trimethyl-ammonium bromide-based extraction method. The T-DNA insertion was identified by PCR with appropriate primers according to SIGnAL (http://signal.salk.edu/cgi-bin/tdnaexpress). The primers for the constructs are listed in Supplemental Table 2.

**Vector Construction and Plant Transformation**

To generate the complementation lines of the *cpk32* mutant, a 3,763-bp genomic DNA fragment containing the *CPK32* coding region and 3′-untranslated region was isolated from the T8H10 BAC clone (ABRC) using *Sal*I and *Ssp*I. This fragment was subcloned into the binary vector pCAMBIA1300. The *CPK32_pro_:GUS* construct was generated by cloning the *CPK32* promoter fragment (1,973 bp) upstream of the *β-GLUCURONIDASE* (*GUS*) coding sequence in the pCAMBIA1381 vector. To generate EF-hand mutant complementation lines, four EF-hands of *CPK32* were mutated on the genomic sequences, respectively, as described previously [43], and the mutated *CPK32* were transformed into *cpk32* knock-down mutant respectively. The construct was introduced into Agrobacterium (*Agrobacterium tumefaciens*) strain GV3101 and then transformed into plants using the floral-dip method as previously described (Clough and Bent, 1998). Homozygous T_3_ transgenic lines were used for further analyses. The primers for the constructs are listed in Supplemental Table 2.

**Analysis of Transcriptional Expression**

Total RNA extraction for RT-PCR was performed according to the manufacturer’s instructions (Trizol reagent, Invitrogen). The primers used to detect the *flc*, *cpk32*, and *cpk32* *flc*, *and cpk32 FCA-OE* plants are listed in Supplemental Table 3. Total RNA for RT-qPCR was extracted from 7-day-old seedlings. First strand cDNAs were synthesized using Superscript II reverse transcriptase according to the manufacturer’s instructions (Invitrogen). RT-qPCR analysis was performed using SYBR Green PCR Master Mix (Applied Biosystems) on an Applied Biosystems 7500 RT-qPCR system. Relative gene expression levels were determined by normalization to *UBQ* using the comparative cycle threshold method (2^–ΔΔCt^).

For RNA gel blot analysis, total RNA was extracted from 7-day-old seedlings germinated on MS medium in LDs. Total RNA (20 μg) was loaded per lane and transferred to a nylon membrane for hybridization (GE Healthcare). The probes were labeled with [α-^32^P] dCTP using random primer labeling reagents (GE Healthcare) and hybridized to the blot. Gene-specific templates were used to generate probes by PCR amplification using the primers listed in Supplemental Table 3. *TUB8* was used as the control gene in this experiment.

**Yeast Two-Hybrid Assay**

The GAL4-based two-hybrid system was used for the yeast two-hybrid assay. The *CPKs* coding sequence was cloned into the pDEST22 vector using the Gateway vector pENTR™/TEV/D-TOPO according to the manufacturer’s instructions (Invitrogen, K2525-20). The *FCA*, *FY*, *FLK*, *FVE*, and *ABF1* coding sequences were cloned into the pDEST32 vector. Yeast strain AH109 was transformed with the bait and prey vectors, and incubated on synthetic dropout medium with dextrose (SD-Leu-Trp) at 28ºC for 3 days to obtain positive clones, which were incubated in (SD-Leu-Trp) solution at 28ºC to an OD_600_ = 1.0. The clones were spotted onto selective (SD-Leu-Trp-His-Ade) and nonselective (SD-Leu-Trp) medium.

**Pull-Down Assays**

To construct the Strep-tag II-CPK32 fusion protein, the full-length *CPK32* coding sequence was amplified using primers that contained the *Strep-tag II* sequence at the 5′-end. The resulting PCR product was cloned into the pCXSN vector driven by *35S* promoter. In this construct, the CPK32 protein sequence was followed by the sequence **WSHPQFEKSGGIEGR** containing the seven-acid Factor Xa restriction protease cleavage site (underlined) as a linker. The construct was introduced into *Agrobacterium* strain GV3101. *Agrobacterium* cultures were infiltrated into the fifth or sixth leaves of ten-leaf-old *N. benthamiana* plants. Plants were grown at 23ºC for 3 days. The fusion protein was extracted as previously described [41].

FCA was divided into N-terminal region (aa Met^-1^–Gln^-438^, named FCA-N, 48 kDa) and C-terminal region (aa Ala^-439^–Ala^-747^, named FCA-C, 34 kDa). The DNA fragments encoding these truncated FCA variants were cloned into pTrc-CKS vector (LikeBio Biotech, Inc., LB-NV). In the resulting constructs, CKS-FCA-N-His/CKS-FCA-C-His were fused with His tag. The proteins were purified according to the manufacturer’s instructions ([www.gelifesciences.com](http://www.gelifesciences.com); Ni Sepharose 6 Fast Flow). The CKS tag was removed using 3C protease and purified again using Ni Sepharose 6 Fast Flow to obtain FCA-N-His/FCA-C-His proteins.

Anti-strep II and anti-His antibodies were used together in the input west-blot assay, and each band can be clearly identified. Strep II-CPK32 was used to pull-down FCA fragments.

**Bimolecular Fluorescence Complementation (BiFC) Assay**

To generate the BiFC vectors, the *CPK32* coding region was cloned via *Bam*HI-*Sal* I into pSPYCE (MR), resulting in *CPK32-YC*. The *FCA* coding region was cloned via *Bam*HI-*Sal*I into pSPYNE(R)173 and pSPYCE (MR), resulting in *YN-FCA* and *FCA-YC*, respectively. The *FY* coding region was cloned via *BamH*I-*Sal*I into pSPYNE(R)173, resulting in *YN-FY*. All constructs were introduced into Agrobacterium strain GV3101. Equal volumes of Agrobacterium cultures harboring *CPK32-YC*, *YN-FCA*, and the viral silencing suppressor P19 were mixed to a final cell density of OD_600_ = 0.8. Agrobacterium cultures were infiltrated into the fifth and sixth leaves of ten-leaf-old Nicotiana *benthamiana* plants*.* Plants were grown at 23ºC for 3 days. DAPI was used to stain the cell nucleus and fluorescence was detected using laser scanning confocal microscope (excitation at 405 nm, and detection between 420 nm and 465 nm). Yellow Fluorescence Protein (YFP) fluorescence (excitation at 488 nm, and detection between 505 nm and 560 nm) was imaged using a laser scanning confocal microscope (Leica STELLARIS5).

**Phosphorylation Assay**

Strep II-CPK32 was purified from tobacco leaves as described in “Pull-down assay”. Phosphorylation assays were performed in 30 μL reaction buffer containing 25 mM Tris-HCI pH 7.5, 10 mM MgCl_2_, 0.5 mM CaCl_2_, 1 mM DTT, and 10 μM ATP. Phosphorylation was initiated by adding 0.5 μCi of [γ-^32^P] ATP, and the reaction was incubated for 20 min at 25ºC. The truncated forms of FCA (FCA-A, FAC-B, FCA-C, FAC-D, FCA-E, FCA-F, FCA-G, and FCA-H) and the mutation were fused with Glutathione S-Transferase (GST), produced in *Escherichia coli* and purified. The protein was separated by 10% SDS-PAGE electrophoresis and the phosphorylated protein was scanned using GE Amersham Typhoon IP.

**ACCESSION NUMBERS**

Sequence data for the genes described in this article can be found in the Arabidopsis Genome Initiative or GenBank/EMBL databases under the following accession numbers: *CPK32* (At3g57530), *FCA* (At4g16280), *FLC* (At5g10140), *FVE* (At2g19520), *FLK* (At3g04610), *FY* (At5g13480), *FLD* (At3g10390), *REF6* (At3g43480), *PRMT5* (At4g31120), and *PRMT10* (At1g04870).
