# Supplementary material for: Calcium-dependent protein kinase CPK32 mediates calcium signaling in regulating Arabidopsis flowering time
Source: Natl Sci Rev. 2021 Sep 27;9(1):nwab180. doi: 10.1093/nsr/nwab180 (PMC8783668; doi:10.1093/nsr/nwab180)
Supplement: nwab180_Supplemental_Files [file nwab180_supplemental_files.zip › Suppl_Figures-cleared.pptx]

## Slide 1
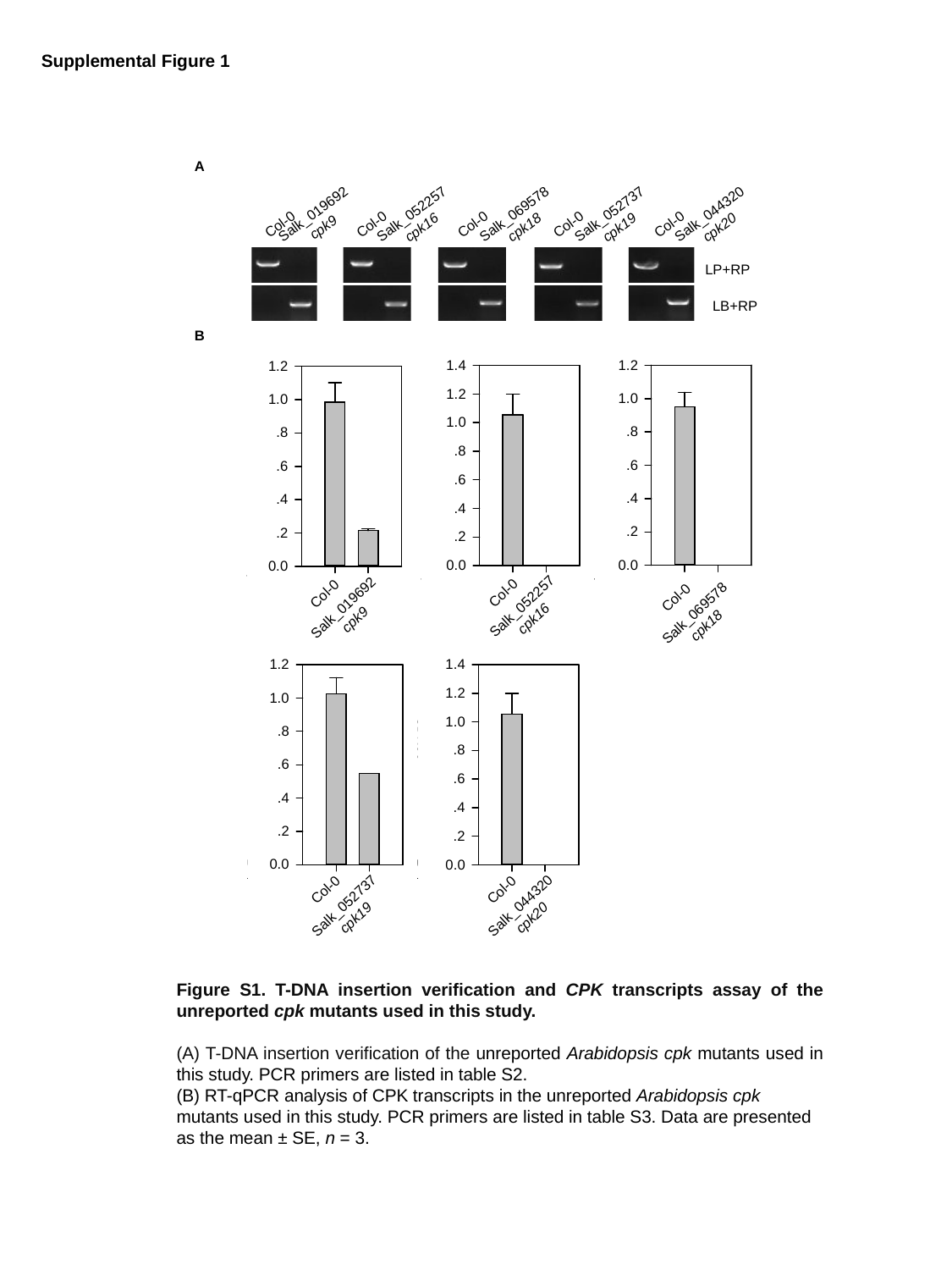

Supplemental Figure 1
A
Salk_019692
cpk9
Col-0
Salk_052257
cpk16
Col-0
Salk_069578
cpk18
Col-0
Salk_052737
cpk19
Col-0
Salk_044320
cpk20
Col-0
LP+RP
LB+RP
B
Col-0
Salk_069578
cpk18
Col-0
Salk_052257
cpk16
Col-0
Salk_019692
cpk9
Col-0
Salk_052737
cpk19
Col-0
Salk_044320
cpk20
Figure S1. T-DNA insertion verification and CPK transcripts assay of the unreported cpk mutants used in this study.
(A) T-DNA insertion verification of the unreported Arabidopsis cpk mutants used in this study. PCR primers are listed in table S2.
(B) RT-qPCR analysis of CPK transcripts in the unreported Arabidopsis cpk mutants used in this study. PCR primers are listed in table S3. Data are presented as the mean ± SE, n = 3.

## Slide 2
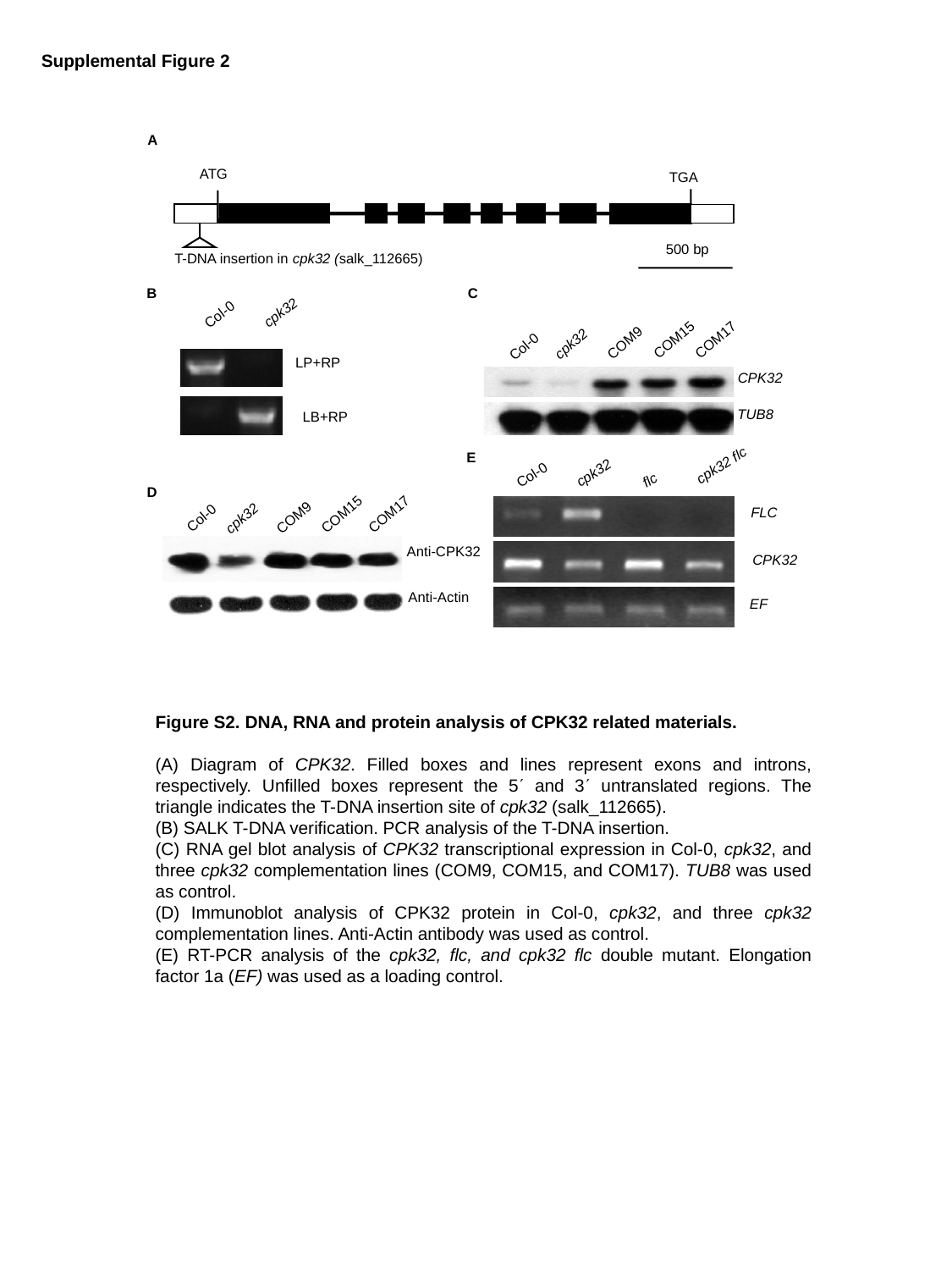

Supplemental Figure 2
A
ATG
TGA
500 bp
T-DNA insertion in cpk32 (salk_112665)
B
cpk32
Col-0
LP+RP
LB+RP
C
COM17
COM15
COM9
cpk32
Col-0
CPK32
TUB8
E
cpk32 flc
cpk32
Col-0
flc
FLC
CPK32
EF
D
COM17
COM15
COM9
cpk32
Col-0
Anti-CPK32
Anti-Actin
Figure S2. DNA, RNA and protein analysis of CPK32 related materials.
(A) Diagram of CPK32. Filled boxes and lines represent exons and introns, respectively. Unfilled boxes represent the 5′ and 3′ untranslated regions. The triangle indicates the T-DNA insertion site of cpk32 (salk_112665).
(B) SALK T-DNA verification. PCR analysis of the T-DNA insertion.
(C) RNA gel blot analysis of CPK32 transcriptional expression in Col-0, cpk32, and three cpk32 complementation lines (COM9, COM15, and COM17). TUB8 was used as control.
(D) Immunoblot analysis of CPK32 protein in Col-0, cpk32, and three cpk32 complementation lines. Anti-Actin antibody was used as control.
(E) RT-PCR analysis of the cpk32, flc, and cpk32 flc double mutant. Elongation factor 1a (EF) was used as a loading control.

## Slide 3
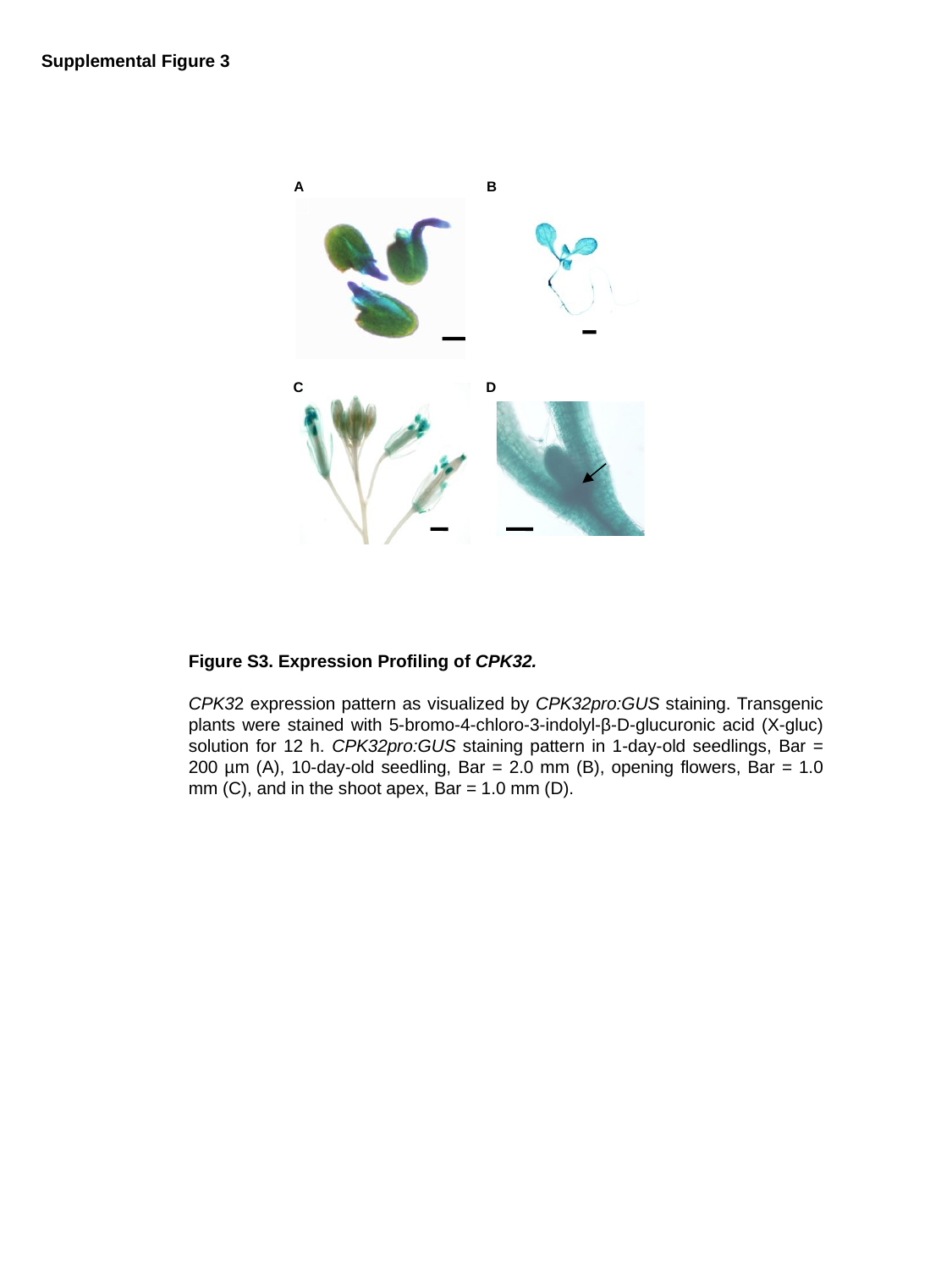

Supplemental Figure 3
B
A
C
D
Figure S3. Expression Profiling of CPK32.
CPK32 expression pattern as visualized by CPK32pro:GUS staining. Transgenic plants were stained with 5-bromo-4-chloro-3-indolyl-β-D-glucuronic acid (X-gluc) solution for 12 h. CPK32pro:GUS staining pattern in 1-day-old seedlings, Bar = 200 µm (A), 10-day-old seedling, Bar = 2.0 mm (B), opening flowers, Bar = 1.0 mm (C), and in the shoot apex, Bar = 1.0 mm (D).

## Slide 4
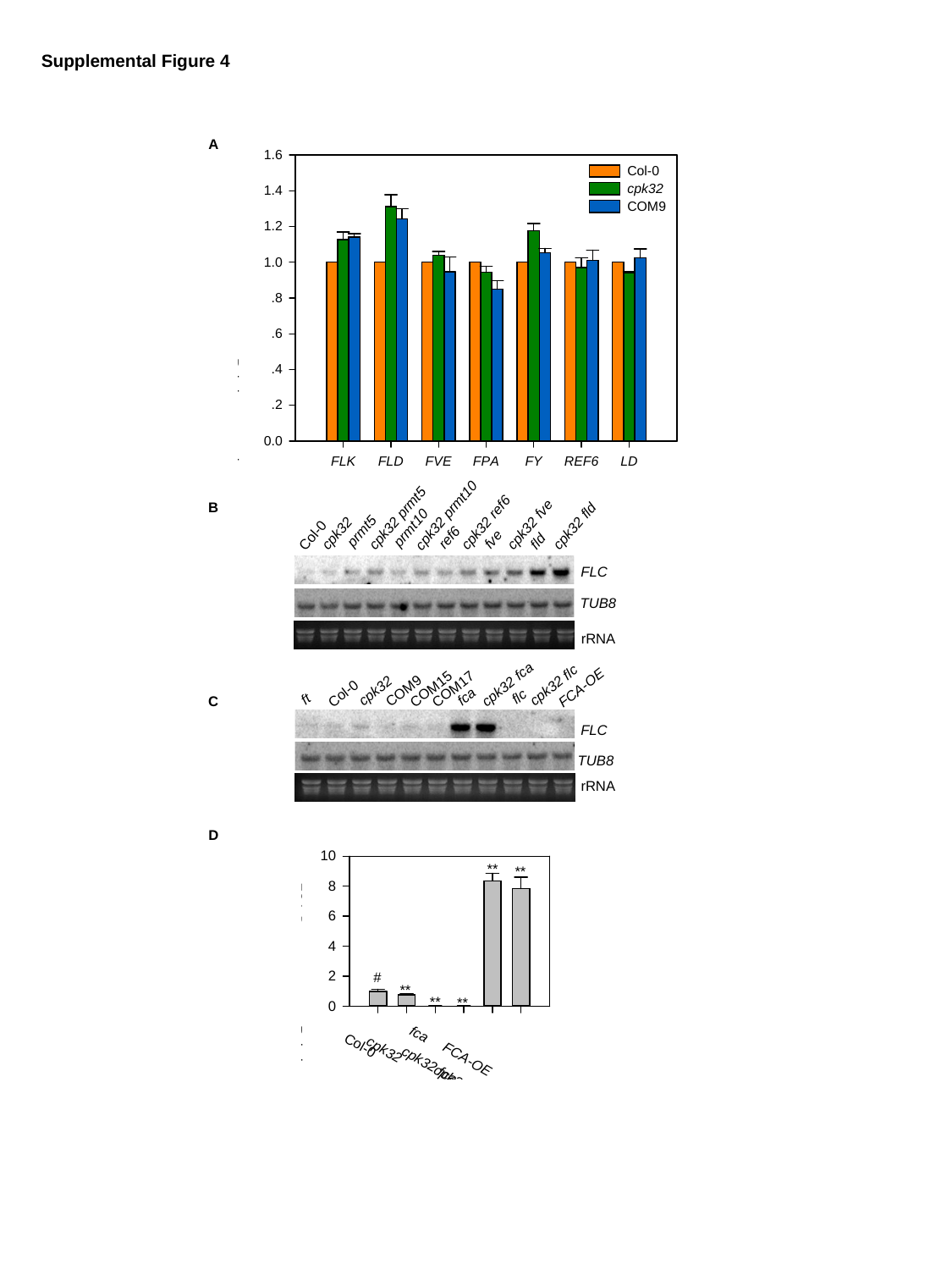

Supplemental Figure 4
A
cpk32 prmt10
cpk32 prmt5
cpk32 ref6
cpk32 fve
cpk32 fld
prmt10
prmt5
cpk32
Col-0
ref6
fve
fld
FLC
TUB8
rRNA
B
cpk32 fca
cpk32 flc
FCA-OE
COM15
COM17
cpk32
COM9
Col-0
fca
flc
ft
FLC
TUB8
rRNA
C
D

## Slide 5
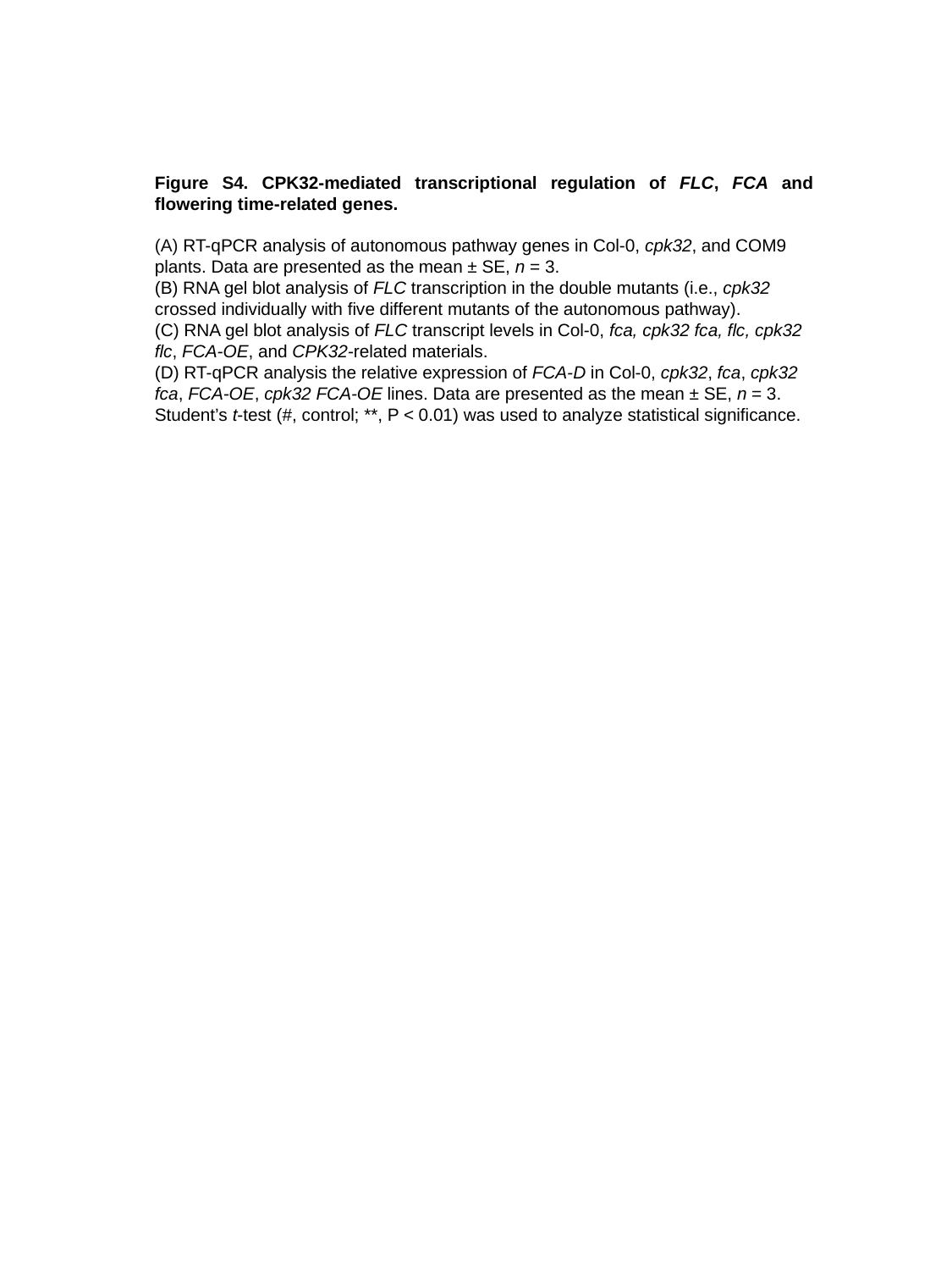

Figure S4. CPK32-mediated transcriptional regulation of FLC, FCA and flowering time-related genes.
(A) RT-qPCR analysis of autonomous pathway genes in Col-0, cpk32, and COM9 plants. Data are presented as the mean ± SE, n = 3.
(B) RNA gel blot analysis of FLC transcription in the double mutants (i.e., cpk32 crossed individually with five different mutants of the autonomous pathway).
(C) RNA gel blot analysis of FLC transcript levels in Col-0, fca, cpk32 fca, flc, cpk32 flc, FCA-OE, and CPK32-related materials.
(D) RT-qPCR analysis the relative expression of FCA-D in Col-0, cpk32, fca, cpk32 fca, FCA-OE, cpk32 FCA-OE lines. Data are presented as the mean ± SE, n = 3. Student’s t-test (#, control; **, P < 0.01) was used to analyze statistical significance.

## Slide 6
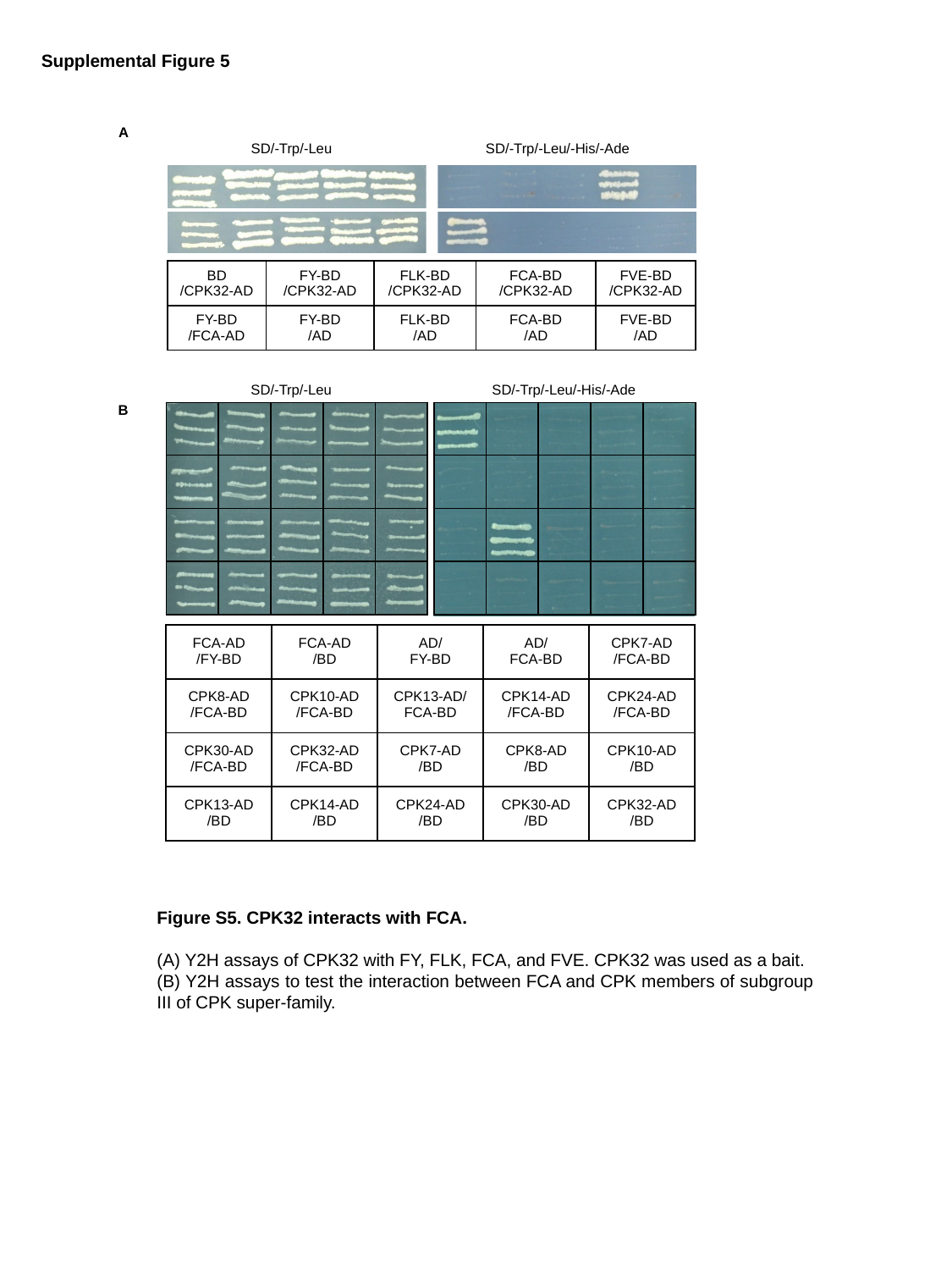

Supplemental Figure 5
A
SD/-Trp/-Leu
SD/-Trp/-Leu/-His/-Ade
| BD /CPK32-AD | FY-BD /CPK32-AD | FLK-BD /CPK32-AD | FCA-BD /CPK32-AD | FVE-BD /CPK32-AD |
| --- | --- | --- | --- | --- |
| FY-BD /FCA-AD | FY-BD /AD | FLK-BD /AD | FCA-BD /AD | FVE-BD /AD |
SD/-Trp/-Leu
SD/-Trp/-Leu/-His/-Ade
B
| | | | | |
| --- | --- | --- | --- | --- |
| | | | | |
| | | | | |
| | | | | |
| | | | | |
| --- | --- | --- | --- | --- |
| | | | | |
| | | | | |
| | | | | |
| FCA-AD /FY-BD | FCA-AD /BD | AD/ FY-BD | AD/ FCA-BD | CPK7-AD /FCA-BD |
| --- | --- | --- | --- | --- |
| CPK8-AD /FCA-BD | CPK10-AD /FCA-BD | CPK13-AD/ FCA-BD | CPK14-AD /FCA-BD | CPK24-AD /FCA-BD |
| CPK30-AD /FCA-BD | CPK32-AD /FCA-BD | CPK7-AD /BD | CPK8-AD /BD | CPK10-AD /BD |
| CPK13-AD /BD | CPK14-AD /BD | CPK24-AD /BD | CPK30-AD /BD | CPK32-AD /BD |
Figure S5. CPK32 interacts with FCA.
(A) Y2H assays of CPK32 with FY, FLK, FCA, and FVE. CPK32 was used as a bait.
(B) Y2H assays to test the interaction between FCA and CPK members of subgroup III of CPK super-family.

## Slide 7
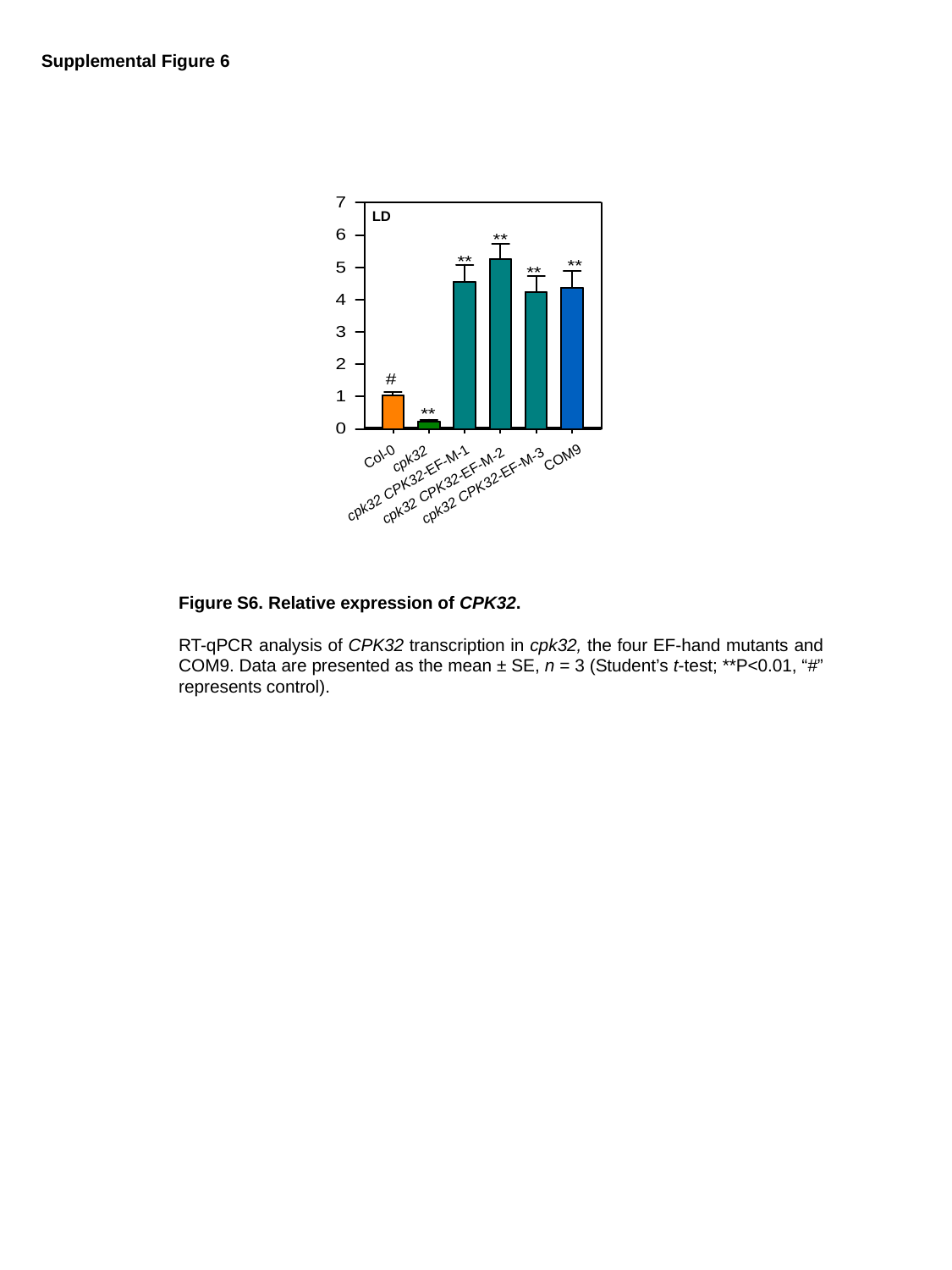

Supplemental Figure 6
LD
Col-0
COM9
cpk32
cpk32 CPK32-EF-M-3
cpk32 CPK32-EF-M-1
cpk32 CPK32-EF-M-2
Figure S6. Relative expression of CPK32.
RT-qPCR analysis of CPK32 transcription in cpk32, the four EF-hand mutants and COM9. Data are presented as the mean ± SE, n = 3 (Student’s t-test; **P<0.01, “#” represents control).
