# Supplementary material for: Calcium-dependent protein kinase CPK32 mediates calcium signaling in regulating Arabidopsis flowering time
Source: Natl Sci Rev. 2021 Sep 27;9(1):nwab180. doi: 10.1093/nsr/nwab180 (PMC8783668; doi:10.1093/nsr/nwab180)
Supplement: nwab180_Supplemental_Files [file nwab180_supplemental_files.zip › Supplemental_tables-cleared.doc]

**Supplemental Table 1. T-DNA insertion lines used in flowering time phenotype screening.**

| AGI | Gene | T-DNA | Gene expression  Knock-down/Knock-out | References/  RT-qPCR analysis |
| --- | --- | --- | --- | --- |
| At5g04870 | CPK1 | SALK_007917 | Knock-out | Qin et al., 2020 |
| At3g10660 | CPK2 | SALK_059237 | Knock-out | Qin et al., 2020 |
| At4g23650 | CPK3 | SALK_107620 | Almost the same as WT | Qin et al., 2020 |
| At4g09570 | CPK4 | SALK_066735 | Knock-down | Qin et al., 2020 |
| At4g35310 | CPK5 | SALK_098331 | Almost the same as WT | Qin et al., 2020 |
| At2g17290 | CPK6 | SALK_025460 | Knock-out | Boudsocq et al., 2010 |
| At5g12480 | CPK7 | SALK_035601 | Knock-out | Liu et al., 2017 |
| At5g19450 | CPK8 | SALK_036581 | Knock-out | Zou et al., 2015 |
| At3g20410 | CPK9 | SALK_019692 | Knock-down | Figure S1 |
| At1g18890 | CPK10 | SALK_082441 | Knock-out | Qin et al., 2020 |
| At1g35670 | CPK11 | SALK_054495 | Knouk-out | Zhu et al., 2007 |
| At1g35670 | CPK12 | SALK_090011 | Almost the same as WT | Qin et al., 2020 |
| At3g51850 | CPK13 | SALK_057893 | Knock-down | Liu et al., 2017 |
| At3g51850 | CPK14 | SALK_100193 | Knock-down | Qin et al., 2020 |
| At2g17890 | CPK16 | SALK_052257 | Knouk-out | Figure S1 |
| At5g12180 | CPK17 | SALK_057146 | Knouk-out | Qin et al., 2020 |
| At4g36070 | CPK18 | SALK_069578 | Knouk-out | Figure S1 |
| At1g61950 | CPK19 | SALK_052737 | Knock-down | Figure S1 |
| At2g38910 | CPK20 | SALK_044320 | Knouk-out | Figure S1 |
| At4g04720 | CPK21 | SALK_029412 | Knouk-out | Franz et al., 2011 |
| At4g04710 | CPK22 | SALK_136629 | Over expression | Qin et al., 2020 |
| At4g04740 | CPK23 | SALK_007958 | Knouk-out | Qin et al., 2020 |
| At2g31500 | CPK24 | SALK_015986 | Knouk-out | Qin et al., 2020 |
| At4g38230 | CPK26 | SALK_107005 | Knouk-out | Qin et al., 2020 |
| At4g04700 | CPK27 | SALK_141648 | Knouk-out | Qin et al., 2020 |
| At5g66210 | CPK28 | SALK_112540 | Over expression | Gao et al., 2014 |
| At1g76040 | CPK29 | SALK_114657 | Knock-down | Qin et al., 2020 |
| At1g74740 | CPK30 | SALK_042171 | Over expression | Liu et al., 2017 |
| At4g04695 | CPK31 | SALK_049228 | Knouk-out | Qin et al., 2020 |
| At3g57530 | CPK32 | SALK_112665 | Knock-down | Figure S2 |
| At1g50700 | CPK33 | SALK_036145 | Knouk-out | Qin et al., 2020 |
| At5g19360 | CPK34 | SALK_040609 | Knouk-out | Qin et al., 2020 |

1. Qin DB, Liu MY and Yuan L *et al*. Calcium-dependent protein kinase 32-mediated phosphorylation is essential for the ammonium transport activity of AMT1;1 in Arabidopsis roots. *J Exp Bot* 2020; **71**: 5087–5097.
2. Boudsocq M, Willmann M, McCormack M *et al*. Differential innate immune signalling via Ca^2+^ sensor protein kinases. *Nature* 2010; **464**: 418-422.
3. Liu KH, Niu Y and Konishi M *et al*. Discovery of nitrate-CPK-NLP signalling in central nutrient-growth networks. *Nature* 2017; **545**: 311–316.
4. Zou JJ, Li XD and Ratnasekera D, *et al*. Arabidopsis CALCIUM-DEPENDENT PROTEIN KINASE8 and CATALASE3 function in abscisic acid-mediated signaling and H_2_O_2_ homeostasis in stomatal guard cells under drought stress. *Plant Cell* 2015; **27**: 1445–1460.
5. Zhu SY, Yu XC and Wang XJ *et al*. Two calcium-dependent protein kinases, CPK4 and CPK11, regulate abscisic acid signal transduction in Arabidopsis. *Plant Cell* 2007; **19**: 3019–3036.
6. Franz S, Ehlert B and Liese A *et al.* Calcium-Dependent Protein Kinase CPK21 functions in abiotic stress response in Arabidopsis thaliana. *Mol Plant* 2011; **4**: 83–96.
7. Gao A, Wu Q and Zhang Y *et al.* Arabidopsis calcium-dependent protein kinase CPK28 is potentially involved in the response to osmotic stress. *Chinese Sci Bull* 2014; **59**: 1113–1122.

**Supplemental Table 2. Primer Sequences Used for T-DNA Insertion Assay and Vector Construction**

| **Primer name** | **gene** | **Primer Sequence (5’-3’)** | **Vector** |
| --- | --- | --- | --- |
| *CPK32-LP* | *CPK32* | ATGCGTTTATCGATTTGTTGG | T-DNA insert assay |
| *CPK32-RP* |  | GAGGTCTTCCACCTCCATCTC |  |
| *CPK9-LP* | *CPK9* | GAGATCGATTTGCAAGCGTAC |  |
| *CPK9-RP* |  | TAGCCAGGCCTAGGAGAAGAG |  |
| *CPK16-LP* | *CPK16* | ATCAATCGCATCAAACTGGTC |  |
| *CPK16-RP* |  | TATGCGAGGGTGGTGAATTAC |  |
| *CPK18-LP* | *CPK18* | TCCAAACGTCTGATTCAGGTC |  |
| *CPK18-RP* |  | GGCCAGTTTGGCTTTACCTAC |  |
| *CPK19-LP* | *CPK19* | GCTCCTTTGATTTCCACAATG |  |
| *CPK19-RP* |  | TCTTCTCAAGGAACAAGACCG |  |
| *CPK20-LP* | *CPK20* | TATCCGCCTGATCAAATGTTC |  |
| *CPK20-RP* |  | CACCGTGATCTTAAGCCTGAG |  |
| *CPK32-OE-F* | *CPK32* | GCTCTAGAATGGGTAATTGTTGCG | pSuper1300 |
| *CPK32-OE-R* |  | CGAGCTCTCATCTTGTATCACCA |  |
| *CPK32-BiFC-ATG-F* |  | GGCTTAAUGGTAATTGTTGCGGAACAGCAGG | pSPYCE(MR) |
| *CPK32-BiFC-R* |  | GGTTTAAUTCATCTTGTATCACCATTGACC |  |
| *CPK32-EF1-M-F* | *CPK32*  *CPK32* | AGGGTTCCAAATAATGGCCACAAGCCAAAGAGGGA | Site mutation  Site mutation |
| *CPK32-EF1-M-R* |  | TCCCTCTTTGGCTTGTGGCCATTATTTGGAACCCT |  |
| *CPK32-EF2-M-F* |  | ATTTTGATGGATGCTGGAGCCATCGCTAGAGATGGATATTTGGAC |  |
| *CPK32-EF2-M-R* |  | GTCCAAATATCCATCTCTAGCGATGGCTCCAGCATCCATCAAAAT |  |
| *CPK32-EF3-M-F* |  | CAAGAAAGCGTTTGCGTTCTTTGCTCAAAACAATAACGGATATATC |  |
| *CPK32-EF3-M-R* |  | GATATATCCGTTATTGTTTTGAGCAAAGAACGCAAACGCTTTCTTG |  |
| *CPK32-EF4-M-F* |  | CCATTATCCGTGATGTTGCCACCGCCAAGGATGGAAGAATAAG |  |
| *CPK32-EF4-M-R* |  | CTTATTCTTCCATCCTTGGCGGTGGCAACATCACGGATAATGG |  |
| CPK32G-e413a/e415a-EF2F |  | CTTCTACTTTGCAGGGAGCCATCGCTAGAGATGGATATTTGGA |  |
| CPK32G-e413a/e415a-EF2R |  | TCCAAATATCCATCTCTAGCGATGGCTCCCTGCAAAGTAGAAG |  |
| CPK32G-EFe484a/e486a-4F |  | CATTATCCGTGATGTTGCCACCGCCAAGGTTGGTTAACTTC |  |
| CPK32G-EFe484a/e486a-4R |  | GAAGTTAACCAACCTTGGCGGTGGCAACATCACGGATAATG |  |
| *FY-BiFC-ATG-F* | *FY* | GGCTTAAUATGCGGCAGTCGT | pSPYNE(R)173 |
| *FY-BiFC-TGA-R* |  | GGTTTAAUCTGATGTTGCTGATTGTTGTTTGG |  |
| *FCA-BiFC-ATG-F* | *FCA* | GGCTTAAUAATGGTCCCCCAGATAGAGTAGAT | pSPYNE(R)173 |
| *FCA-BiFC-TGA-R* |  | GGTTTAAUAGCTTTATTCTTCCACATGAGTTC |  |
| *CPK32-pDEST22-F* | *CPK32* | CACCATGGGTAATTGTTGCG | pDEST22 |
| *CPK32-pDEST22-R* |  | TCATCTTGTATCACCATTGACC |  |
| *FCA-pDEST32-F* | *FCA* | CACCATGGGAACTCACATCAATT | pDEST32 |
| *FCA-pDEST32-R* |  | CTAACCCATCTGTTGAACACAGTTATC |  |
| *ABF1-pDEST32-F* | *ABF1* | CACCATGGGTACTCACATTGATATC | pDEST32 |
| *ABF1-pDEST32-R* |  | TCATTCGTTCTGCTGAGTATTATCT |  |
| *FY-pDEST32-F* | *FY* | CACCATGATGCGGCAGTCGTCAGCTT | pDEST32 |
| *FY-pDEST32-R* |  | CTACTGATGTTGCTGATTGTTGTTTGG |  |
| *FLK-pDEST32-F* | *FLK* | CACCATGGCTGAAGCTGAAGATC | pDEST32 |
| *FLK-pDEST32-R* |  | TCAGTAACCGTAGCCTGAGCTGTAATC |  |
| *FVE-pDEST32-F* | *FVE* | CACCATGGAGAGCGACGAAGC | pDEST32 |
| *FVE-pDEST32-R* |  | TTAAGGCTTGGAGGCACAAGTCATA |  |
| *Strep-CPK32-F* | *CPK32* | GATGTGGTCTCATCCTCAATTTGAAAAAAGCGGCGGCATCGAAGGTCGTGGTAATTGTTGCGGAACAGCAGG | pCXSN |
| *FCA-CKS-N-R* | *FCA* | TCTAGACTGGGCCCCAGGGAAATTAGTTTGC | pTrc-CKS |
| *FCA-CKS-C-F* |  | GAATTCGCACCCTTGCAGAATCCTTATGCTT |  |
| *USER-FCA-584-F* | *FCA* | GGCTTAAUCCTGCAGTAAGTCAGAGCGTAGGTTC | pGEX-4T-1 |
| *USER-FCA-591-F* |  | GGCTTAAUGGTTCTGTCAAATGTACCTGGACC |  |
| *USER-FCA-624-F* |  | GGCTTAAUGAAATGATAGTGTTCGAACGAGAGC |  |
| *USER-FCA-624-R* |  | GGTTTAAUTTCCTCAGGTTTTTCCCACTTGCTT |  |
| *USER-FCA-747-R* |  | GGTTTAAUAGCTTTATTCTTCCACATGAGTTCTTG |  |
| *FCA^S587A^-F* | *FCA*  *FCA* | CAGCTGCACCTGCAGTAGCTCAGAGCGTAGGTTC | Site mutation  Site mutation |
| *FCA^S587A^-R* |  | GAACCTACGCTCTGAGCTACTGCAGGTGCAGCTG |  |
| *FCA^S589A^-F* |  | CATTTGACAGAACCTACGGCCTGACTTACTGCAGGTGC |  |
| *FCA^S589A^-R* |  | GCACCTGCAGTAAGTCAGGCCGTAGGTTCTGTCAAATG |  |
| *FCA^S592A^-F* |  | CCAGGTACATTTGACAGCACCTACGCTCTGACTTA |  |
| *FCA^S592A^-R* |  | TAAGTCAGAGCGTAGGTGCTGTCAAATGTACCTGG |  |
| *FCA^S592D^-F* |  | GTCCAGGTACATTTGACATCACCTACGCTCTGACTTACTG |  |
| *FCA^S592D^-R* |  | CAGTAAGTCAGAGCGTAGGTGATGTCAAATGTACCTGGAC |  |
| *FCA^T596A^-F* |  | CTCGGTCCAGGCACATTTGACAGAACCTACGCT |  |
| *FCA^T596A^-R* |  | AGCGTAGGTTCTGTCAAATGTGCCTGGACCGAG |  |
| *FCA^T598A^-F* |  | GCGAGGTATGCTCGGCCCAGGTACATTTGAC |  |
| *FCA^T598A^-R* |  | GTCAAATGTACCTGGGCCGAGCATACCTCGC |  |
| *FCA^S601A^-F* |  | TCAGGCGAGGCATGCTCGGTCCAGGTACA |  |
| *FCA^S601A^-R* |  | TGTACCTGGACCGAGCATGCCTCGCCTGA |  |
| *FCA^T602A^-F* |  | TTAAATCCATCAGGCGCGGTATGCTCGGTCCAG |  |
| *FCA^T602A^-R* |  | CTGGACCGAGCATACCGCGCCTGATGGATTTAA |  |
| *FCA^S617A^-F* |  | CTCAGGTTTTTCCCACTTGGCTTCACCCGTTAGACCATTG |  |
| *FCA^S617A^-R* |  | CAATGGTCTAACGGGTGAAGCCAAGTGGGAAAAACCTGAG |  |
| *FCA^Y614A^-F* |  | TCCCACTTGCTTTCACCCGCTAGACCATTGTAATAATATTTAAATC |  |
| *FCA^Y614A^-R* |  | GATTTAAATATTATTACAATGGTCTAGCGGGTGAAAGCAAGTGGGA |  |
| *FCA^Y608A^-F* |  | CACCCGTTAGACCATTGTAATAAGCTTTAAATCCATCAGGCGAGGTATG |  |
| *FCA^Y608A^-R* |  | CATACCTCGCCTGATGGATTTAAAGCTTATTACAATGGTCTAACGGGTG |  |
| *FCA^Y609A^-F* |  | ACCCGTTAGACCATTGTAAGCATATTTAAATCCATCAGGCGAGGTATG |  |
| *FCA^Y609A^-R* |  | CATACCTCGCCTGATGGATTTAAATATGCTTACAATGGTCTAACGGGT |  |
| *FCA^Y610A^-F* |  | CACCCGTTAGACCATTGGCATAATATTTAAATCCATCAGGCGAGGTATG |  |
| *FCA^Y610A^-R* |  | CATACCTCGCCTGATGGATTTAAATATTATGCCAATGGTCTAACGGGTG |  |

**Supplemental Table 3. Primer Sequences Used for RNA analysis.**

| **gene** | **Primer name** | **Primer Sequence (5’-3’)** |
| --- | --- | --- |
| **RT-PCR** | | |
| *CPK32* | *CPK32-F* | ATGGGTAATTGTTGCG |
|  | *CPK32-R* | TCATCTTGTATCACCA |
| *FLC* | *FLC-F* | TGTTCTCTGTGACGCATCC |
|  | *FLC-R* | ACCTGGTTCTCTTCTTTCAGC |
| *EF1α* | *EF1α-F* | ATGCCCCAGGACATCGTGATTTCAT |
|  | *EF1α-R* | TTGGCGGCACCCTTAGCTGGATCA |
| **RT-qPCR** | | |
| *FLC* | *FLC-F* | CGATGCTCTTGTTCAACTGG |
|  | *FLC-R* | CTTCTGCTCCCACATGATGA |
| *UBQ10* | *UBQ10-F* | GGGCACTCAAGTATCTTGTTAGC |
|  | *UBQ10-R* | TGCTGCCCAACATCAGGTT |
| *FCA-P* | *FCA-P-F* | GGCAGCTTTACTGGAACTGATGTCTC |
|  | *FCA-P-R* | GCTTCCCATCAGCCTTTTCAGGGAGAC |
| *FCA-D* | *FCA-D-F* | TGGGAAAAACCTGAGGAAAT |
|  | *FCA-D-R* | CAGTGAAGAATAAAACGGCT |
| *LD* | *LD-F* | CAACTAATCCTGGAATGAGTGG |
|  | *LD-R* | GGTTGTTGAGATTGGTTGTTGT |
| *FY* | *FY-F* | GATGCCTGGATCAATGGGAATG |
|  | *FY-R* | TGCTGCTGTTGGAAAGGGTTGT |
| *FLK* | *FLK-F* | CCACCAATGGTCGCTCAGCAAG |
|  | *FLK-R* | ATCCGTAGCGTATCCTCCAGGCG |
| *FLD* | *FLD-F* | TGCGGGAGAAGCTGCTCATAA |
|  | *FLD-R* | GCCTTTGCAGATTGAGCCATG |
| *FVE* | *FVE-F* | ATTCAGCCCGACGAGTGCACA |
|  | *FVE-R* | CTGCAATGTTCCACCTCCACC |
| *REF6* | *REF6-F* | CGGAATACCGTGTTGCAGGTTAG |
|  | *REF6-R* | CCGGATAGCAGCATCTTTAGCCA |
| *FPA* | *FPA-F* | TGGGTGTCGATGAGAGGTCAT |
|  | *FPA-R* | CAATTGACGACCTCAGGCAGT |
| *CPK9* | *CPK9-F* | GCGAACAAACCAAGAACCAGATG |
|  | *CPK9-R* | TCAGACATTCATAGACTCATCAGGAAGA |
| *CPK16* | *CPK16-F* | GGTCTCTGTTTCTCCTCCGCC |
|  | *CPK16-R* | TTAGACCTTGCGAGAAATAAGATAACC |
| *CPK18* | *CPK18-F* | GGTCTCTGTTTCTCGTCTCCT |
|  | *CPK18-R* | TTATAAGACTTGAGAAACATATTCAAACCTAACGG |
| *CPK19* | *CPK19-F* | GGATGTCTCTGCATCAATCTGAAG |
|  | *CPK19-R* | TTAATTCGGTTGAACCAATTTTGATTGG |
| *CPK20* | *CPK20-F* | GGAAACACATGCGTGGGTCCG |
|  | *CPK20-R* | TCAACTCACCTTTAAACCCATTTTGCC |
| *FT* | *FT-F* | GCTTGGATCTAAGGCCTTCTCA |
|  | *FT-R* | TGGGACTTGGATTTTCGTAACA |
| *SOC1* | *SOC1-F* | TTGTTGAAGAAAGCCTTTGAGC |
|  | *SOC1-R* | TTTGGTGCTGACTCGATCC |
| **Northern Blot** | | |
| *CPK32* | *CPK32-F* | ATGCCTGAGCATCCTAA |
|  | *CPK32-R* | TCATCTTGTATCACCATTGACC |
| *FLC* | *FLC-F* | TCTCCTCCGGCGATAACCT |
|  | *FLC-R* | GCATGCTGTTTCCCATATCGAT |
| *TUB8* | *TUB8-F* | CTCAAGAGGTTCTCAGCAGTA |
|  | *TUB8-R* | TCACCTTCTTCATCCGCAGTT |
